# Key and evolving needs of service providers in women’s harm reduction centers during the COVID-19 pandemic

**DOI:** 10.1186/s12954-025-01228-6

**Published:** 2025-04-25

**Authors:** Azam Rahmani, Maryam Janatolmakan, Elham Rezaei, Malihe Tabarrai

**Affiliations:** 1 Nursing and Midwifery Care Research Center, School of Nursing and Midwifery, Tehran University of Medical Sciences, Tehran, Iran; 2 Social Development and Health Promotion Research Center, Health Institute, Kermanshah University of Medical Sciences, Kermanshah, Iran; 3 Nursing and Midwifery Care Research Center, Tehran University of Medical Sciences, Tehran, Iran; 4 Midwifery Department, School of Nursing and Midwifery, Clinical Research Development Unit, Talegani Hospital, Tabriz University of Medical Sciences, Tabriz, Iran; 5 Department of Persian Medicine, School of Persian Medicine, Tehran University of Medical Sciences, Tehran, Iran

**Keywords:** COVID-19, Harm reduction, HIV, Iran, Qualitative study, Providers

## Abstract

**Background:**

Service providers at women’s harm reduction centers maintained direct contact with their clients throughout the COVID-19 pandemic, providing valuable insights into their evolving needs. This study aimed to explore these emerging needs from the perspective of service providers.

**Methods:**

This qualitative study utilized conventional content analysis. Data were gathered through semi-structured face-to-face interviews conducted with ten service providers employed in women’s harm reduction centers situated across three Iranian provinces (Tehran, Khuzestan, and Kermanshah) between January and May 2023. Participant selection was guided by purposive sampling, specifically utilizing Maximum Variation Sampling.

**Results:**

The findings of this study showed that service providers specifically need to alleviate credit constraints and control inflation. Additionally, the necessity for continuous monitoring, revision of evaluative criteria, centralization of shelters and drop-in centers, and adequate staffing was emphasized. The need for flexible regulations, clear job descriptions, improved accommodations, and sufficient equipment was also highlighted. Employee safety and effective judicial protection were among other important needs. This study also emphasized the need to change negative social attitudes and enhance professional development for social workers. Developing creative educational approaches and conducting rigorous needs assessments were also among the significant findings. These findings can help policymakers design more effective support programs for service providers and improve service quality by providing appropriate communication tools.

**Conclusion:**

The findings of this study underscore the critical importance of policymakers addressing this spectrum of identified needs to ensure the effective delivery of harm reduction services for women, not only during the COVID-19 pandemic but also in similar future crises.

## Introduction

Women’s harm reduction centers play a crucial role in supporting vulnerable populations, including homeless women, sex workers, individuals living with HIV, and people who use drugs. These centers offer essential services such as distributing harm reduction supplies (e.g., clean syringes, condoms), conducting screenings and vaccinations, providing counseling, and offering basic necessities like food and shelter [1, 2]. However, the COVID-19 pandemic significantly disrupted the delivery of these essential services [3]. Service providers in these centers, who are on the frontline, experienced firsthand the challenges and adaptations necessary to continue supporting their clients during this unprecedented public health crisis [4].

Previous research has documented the challenges faced by service providers during the COVID-19 pandemic. For instance, studies in Denmark highlighted the critical need for inter-organizational collaboration and innovative approaches like mobile testing units and temporary shelters [5]. Similarly, research identified significant barriers such as misconceptions about COVID-19 and limited access to essential resources [6]. In the United States, homeless service providers faced numerous individual and organizational challenges while demonstrating resilience and creativity in developing solutions [7]. These international studies [5–7] underscore the broad impact of the pandemic on service provision and the adaptations required.

Despite significant international research examining the experiences of service providers in various sectors affected by the COVID-19 pandemic, such as harm reduction and HIV treatment for people who inject drugs and alcohol [8, 9], individuals experiencing homelessness [10, 11], and general HIV services [12], a thorough and specific investigation into the experiences of service providers within women’s harm reduction centers in Iran is noticeably absent from current literature. These international studies, while informative, do not address the unique context of these specific centers in Iran. Studies conducted within Iran have predominantly investigated the provision of HIV-related services in provinces such as Tehran, Khorasan Razavi, and Kerman [13] from the perspectives of policymakers, service administrators, and service recipients. Furthermore, some research in Tehran and Tabriz has addressed the role of non-governmental organizations (NGOs) in this area [14–16].

In spite of these valuable contributions, a crucial gap persists regarding the specific perspectives and experiences of service providers working within governmental women’s harm reduction centers in Iran. These governmental centers play a unique and critical role by offering a comprehensive spectrum of services to highly vulnerable populations, encompassing homeless women, individuals who inject drugs, and people living with HIV– often with specific mandates and operational contexts distinct from NGOs. Therefore, the present study seeks to address this identified research gap by focusing specifically on this cohort of service providers within these comprehensive governmental centers. Our aim is to gain a more in-depth understanding of their key and evolving needs during the COVID-19 pandemic. This focused approach is anticipated to yield valuable insights that can inform more effective policymaking and the delivery of targeted and responsive services in future public health crises.

## Materials and methods

### Study design

This qualitative study employed conventional content analysis, an inductive approach suitable for exploring participants’ subjective experiences within a relatively novel research area. This method involves an iterative process of deriving codes directly from the collected data, allowing for the emergence of themes grounded in the participants’ own perspectives [17].

### Research sites

This study was conducted in women-specific harm reduction centers located in the provinces of Tehran, Khuzestan, and Kermanshah, Iran. This selection was strategically informed by several key factors beyond mere geographical diversity. Firstly, these locations represent distinct geographical regions of Iran– Tehran Province as the central region, Khuzestan Province in the southwest, and Kermanshah Province in the west– allowing for the capture of potential regional variations in service delivery, client needs, and organizational responses to the COVID-19 pandemic. Secondly, Tehran and Khuzestan Provinces hold significant prominence in the national landscape of harm reduction services for women, hosting a substantial proportion of such centers and serving a diverse clientele with varying socio-economic backgrounds and substance use patterns. Kermanshah Province was also included to capture the experiences in the western region. Their long operational history in providing these specialized services ensures a wealth of experience and established protocols that were likely impacted by the pandemic. Finally, while other provinces also have women’s harm reduction centers, the provinces of Tehran, Khuzestan, and Kermanshah were chosen to provide a manageable scope for this in-depth qualitative study while still capturing a meaningful cross-section of the national landscape based on the aforementioned criteria of geographical spread, service volume, and established presence in the field.

### Study population, sampling, and recruitment

This study focuses on service providers working in women’s harm reduction centers located in Tehran, Khuzestan, and Kermanshah, three major cities representing diverse geographical and socio-economic contexts within Iran. The target population included all service providers with substantial experience working with vulnerable populations, specifically those employed in these centers before and during the COVID-19 pandemic.

To comprehensively understand the evolving needs of service providers in women’s harm reduction centers during the COVID-19 pandemic, a rigorous purposive sampling strategy, specifically Maximum Variation Sampling, was employed. The decision to include ten participants was based on the principle of data saturation within this specific context. These participants were carefully selected from the centers due to their extensive experience providing services before, during, and after the COVID-19 pandemic. Given their comprehensive firsthand knowledge, we anticipated reaching data saturation– the point at which new interviews would not yield significant additional insights– with this number of participants. We aimed to capture the diverse perspectives within these experienced frontline service providers, and the final sample of ten was deemed sufficient to achieve a thorough exploration of the research questions, particularly considering the depth of their individual experiences and the consistency of emerging themes during the data collection process. In fact, in the last interview, we did not achieve any new data.

Semi-structured interviews were conducted in a quiet and private setting at times convenient for participants. Interview questions explored topics such as budget fluctuations, changes in client numbers, COVID-19 prevalence among clients or staff, related social crises, and shifts in harm reduction policies. To delve deeper and clarify concepts, probing questions like “Please explain,” “Why,” and “How” were used.

Data collection took place between January and May 2023. Inclusion criteria for participation were:

Willingness to participate in the study.

Experience working in women’s harm reduction centers before and during the COVID-19 pandemic.

### Sample size

Data saturation was employed as the guiding principle for determining sample size [18]. Data collection proceeded sequentially through in-depth interviews until no novel codes or themes emerged from the data. This iterative process involved ongoing data analysis and comparison of interview transcripts to identify recurring patterns and unique responses. Researchers engaged in regular team meetings and discussions to collaboratively assess data saturation and ensure the comprehensiveness and validity of the collected data. Saturation was achieved after conducting interviews with 10 participants. To further strengthen the rigor of our sampling strategy, Maximum Variation Sampling was employed.

### Data analysis

Data analysis was conducted using the six-step approach outlined by Graneheim and Lundman (2004). This approach involved: (1) conducting and transcribing all interviews verbatim; (2) conducting an initial comprehensive reading of all transcripts to gain an overall understanding of the data; (3) identifying meaning units within the text; (4) assigning initial codes to these meaning units; (5) categorizing these primary codes into broader categories; and (6) identifying overarching themes and subthemes within the data [19]. MAXQDA software (version 10) was utilized for data management, facilitating the organization and analysis of the qualitative data.

### Trustworthiness

To establish the trustworthiness of our findings, we adopted the criteria for trustworthiness proposed by Lincoln and Guba (1985), encompassing validity, transferability, dependability, and confirmability [20]. This approach emphasizes four key criteria including credibility, dependability, confirmability and transferability. In this regard, to ensure that the data accurately reflects the participants’ experiences, member checking, prolonged engagement, and peer debriefing occurred. To ensuring the stability and consistency of the findings, an audit trail was maintained. To ensure the objectivity of the findings and to minimize researcher bias, a confirmability audit was conducted by an external reviewer. To ensuring the applicability of the findings, careful description and purposive sampling was done.

In this study, to manage researcher bias and enhance trustworthiness, reflexivity was employed. Reflexivity refers to researchers utilizing their self-awareness and understanding of how they influence the research process [21]. In this study, researchers, aware of potential biases, endeavored to maintain objectivity and neutrality in data collection and analysis to achieve objective results. Additionally, to reduce bias, multiple researchers were used to review and validate the findings to ensure that the results were obtained independently and without the influence of individual biases.

### Ethical considerations

The research followed the code of ethics from the Ethics Committee of Tehran University of Medical Sciences (IR.TUMS.FNM.REC.1401.175). The study objectives were thoroughly explained to all participants. Prior to data collection, written informed consent was obtained from each participant, emphasizing the confidentiality of their personal information and statements.

## Results

### Participant characteristics

The study included 10 service providers working in women’s harm reduction centers across three major cities in Iran: Tehran, Ahvaz, and Kermanshah. The average age of the participants was 38.0 ± 4.0 years. The majority of participants were women (*n* = 9, 90.0%). More than half of the participants held a bachelor’s degree (*n* = 7, 70.0%), and 60.0% (*n* = 6) were single. All participants were employed informally within the harm reduction centers. (Table 1)

**Table 1:** Demographic characteristics of participants (*n* = 10)

Variables		*N*(%)^+^
Age (mean ± SD)^++^		38.0 ± 4.0
Gender	Female	9(90.0)
	Male	1(10.0)
Marital status	Single	7(70.0)
	Married	3(30.0)
Education status	Diploma	2(20.0)
	Bachelor	6(60.0)
	Master	2(20.0)
Employ status	Unofficial	10(100.0)
	Official	0(0.0)

^+^ Number (percentage); ^++^ Mean ± standard deviation

### Main theme: key and evolving needs of service providers

Based on the in-depth interviews, several Key and Evolving Needs were identified for service providers working in women’s harm reduction centers during the COVID-19 pandemic.

These demands were categorized into seven broad areas: financial needs, executive needs, structural needs, civil needs, social-cultural needs, educational needs, and communication needs (Table 2).

**Table 2:** Key and evolving needs of harm reduction providers during the COVID-19 pandemic

Categories	Subcategories
Financial needs	Removing credit limits
	Controlling inflation
Executive needs	Continuous monitoring
	Revision of the evaluative criteria
Structural needs	Co-location of shelters and DICs *
	Adequate staffing
	Flexible regulations
	Clear job descriptions
	Improving accommodation
	Sufficient equipment
Civil needs	Ensuring employee safety
	Effective judicial protection
Social-cultural needs	Changing negative social attitudes
	Enhancing professional development for social workers
Educational needs	Developing creative training approaches
	Conducting rigorous needs assessments
Communication needs	Behavioral therapy
	Communication tools

*Drop-in center

### Financial needs

Financial and credit needs, already impacting activities, were significantly intensified during the COVID-19 pandemic. This period highlighted the crucial sub-categories of “removing credit limits” and “controlling inflation”.

#### Removing credit limits

Participants emphasized the heightened need for increased government funding during the COVID-19 pandemic to support the implementation of enhanced health and safety protocols, including prevention, screening, vaccination, and client education programs. This requirement, while present before, was significantly intensified by the demands of the pandemic response.

*“The pandemic intensified the financial strain on our centers; the lack of budget and available credit made managing prevention expenses incredibly challenging. Removing credit limits would have been a vital step.“(Participant 1)*.

*“Despite receiving specific government funding for our services*,* harm reduction centers lacked the financial resources to cover the additional preventive*,* health*,* and educational services necessitated by the COVID-19 pandemic.“(Participant 6)*.

#### Controlling inflation

The continuous and general increase in the price of personal protective equipment (PPE) and screening supplies to adhere to health protocols during the COVID-19 pandemic was a concern that participants highlighted the need to curb and control inflation during that period.

*“Despite public support and the government funding allocated to centers*,* managing the increased costs of equipment and supplies for providing services was very difficult*,* which further emphasized the need to control inflation.” (Participant 9)*.

*“"Inflation has long been an issue in Iran*,* but the pandemic acutely highlighted this problem for our centers*,* making its control even more critical.” (Participant 10)*.

### Executive needs

The participants underscored the significance of effective supervision of executive operations within centers to enhance the process of providing services, particularly throughout the COVID-19 pandemic. Two key aspects were highlighted from their viewpoint: “continuous monitoring” and “revising evaluation standards.

#### Continuous monitoring

The unforeseen challenges stemming from the COVID-19 pandemic exerted a detrimental impact across all facets of harm reduction centers, including the well-being of service providers. Consequently, participants underscored the imperative for enhanced and more consistent oversight, identifying it as a salient necessity.

*“During the COVID-19 pandemic*,* our salaries were often late due to managers’ concerns about managing the illness. We wish there had been more oversight to ensure the timely payment of employee salaries and benefits.” (Participant 2)*.

*“I think if officials had provided more continuous oversight during the COVID-19 pandemic*,* it could have significantly reduced the challenges our centers faced.” (Participant 5)*.

#### Revision of evaluative criteria

Participants emphasized the need for a more nuanced and empathetic approach to performance evaluation, considering the unique challenges faced by service providers during the pandemic.

*“During the COVID-19 pandemic*,* assessors at harm reduction centers imposed excessive pressure and stress on us with their rigid and inflexible enforcement. We wish that the evaluation criteria had been revised during that critical time.“(Participant 2)*.

*“In my opinion*,* sticking to a rigid evaluation method during crises such as the COVID-19 pandemic is a serious error. We need to recognize that the circumstances for both the centers and the people they serve are no longer what they used to be.” (Participant 7)*.

### Structural needs

To improve how they deliver services, participants identified several underlying needs for harm reduction centers. These involve co-location of shelters and DICs, adequate staffing, flexible regulations, clear job descriptions, improving accommodation, and sufficient equipment. The COVID-19 pandemic highlighted how crucial it is to address these structural needs, as they were vital for providing effective services during a crisis.

#### Co-location of shelters and DICs

During the COVID-19 pandemic, participants identified communication barriers, including traffic congestion and transportation costs, as significant obstacles to client access. They proposed the co-location of shelters and DICs as a potential solution to address these logistical challenges.

*“During the COVID-19 pandemic*,* some clients were hesitant to access essential education and prevention services due to fears of COVID-19 infection. By integrating shelters and DICs within harm reduction centers*,* client access would have improved*,* and our more effective oversight would have been possible.“(Participant 5)*.

#### Adequate staffing

Participants at harm reduction centers identified specific factors as the reasons for their staff shortages. These key factors included delayed payments, difficult job descriptions, uncompetitive wages, and generally low morale. During the COVID-19 pandemic, these problems not only intensified but also became more pronounced and evident.

*“The COVID-19 pandemic and the resulting staff shortages led to a heavy workload for us*,* a burden that could have been eased by hiring more service providers.” (Participant 7)*.

*“Due to COVID-19 anxieties*,* fewer colleagues remained*,* and contract non-renewals intensified the urgency for recruitment.” (Participant 10)*.

#### Flexible regulations

Participants stressed the need for flexible regulations during the COVID-19 pandemic, recognizing its significance in facilitating the faster delivery of essential services.

*“Harm reduction centers serve a specific population with unique needs*,* necessitating flexible regulations. Unfortunately*,* this need for flexibility was often overlooked during the COVID-19 pandemic.” (Participant 2)*.

“*The full implementation of health procedures*,* such as maintaining social distancing and mask-wearing*,* presented challenges with our clients. Given our target demographic*,* I believe these regulations could have been more flexible.” (Participant 9)*.

#### Clear job descriptions

Service providers highlighted a significant expansion of their responsibilities during the COVID-19 pandemic, many of which were not explicitly outlined in their original job descriptions.

“*The COVID-19 pandemic led to a much larger scope of work for us*,* and we found ourselves performing tasks outside our original job descriptions. We really wish we had had clear and defined job descriptions during that time.” (Participant 7)*.

#### Improving accommodation

The COVID-19 pandemic urgently highlighted the necessity for increased accommodation space in women’s harm reduction centers to effectively implement health protocols and social distancing measures. Participants emphasized this critical need, stating that….

*“During the COVID-19 pandemic*,* the necessity of developing more accommodation space became apparent to us*,* as we lacked sufficient and suitable areas to quarantine clients suspected of having COVID-19.” (Participant 9)*.

#### Sufficient equipment

Participants reported an increase in the number of clients and misuse of facilities during COVID-19, leading to equipment shortages and accelerated equipment wear and tear. Daily cleaning and disinfection also increased the need for resources.

*" The showers*,* bathroom handles*,* and toilets in our center require frequent repairs*,* breaking down approximately every two months. This issue became more pronounced during COVID-19 due to increased usage.” (Participant 6)*.

*" Equipment shortages have long been a problem for harm reduction centers*,* a problem that was exacerbated by the implementation of COVID-19 safety protocols for staff and clients”. (Participant 9)*

### Civil needs

Throughout the COVID-19 pandemic, service providers at harm reduction centers for women highlighted the increasing significance of respecting their civil rights. Their statements identified “ensuring employee safety” and “effective judicial protection” as key and interconnected needs in this regard.

#### Ensuring employee safety

Security is a prerequisite for a peaceful existence. Participants highlighted the heightened need for security during the COVID-19 pandemic to ensure the delivery of quality services to their clientele. One participant noted that….

*“Implementing health guidelines during COVID-19 proved challenging due to interactions with some patients experiencing mental illness. These interactions posed a safety risk for our staff. I hope authorities will address this issue to ensure our peace of mind.“(Participant 4)*.

#### Effective judicial protection

Participants referred to the increased need for enhanced legal and judicial protections to address challenges posed by specific clients during the COVID-19 pandemic, compared to before.

*“Harm reduction service providers have long needed legal support*,* a need intensified by the unique challenges of the COVID-19 pandemic.” (Participant 3)*.

*“During the COVID-19 pandemic*,* if any incidents involving our customers occurred*,* we were required to report them to legal authorities*,* including the judiciary and center managers. The lack of clear legal guidelines and adequate judicial support in these situations made us feel insecure and vulnerable.” (Participant 8)*.

### Social-cultural needs

During the COVID-19 pandemic, the social and cultural needs of service providers at harm reduction centers became even more apparent. This was clearly highlighted in participant statements, particularly concerning “changing negative social attitudes” and “enhancing professional development for social workers.”

#### Changing negative social attitudes

Harm reduction centers and their clients face significant social stigma. This stigma was particularly evident during the COVID-19 pandemic, as many people believed they had contributed to the spread of the disease. As a result, the need to change public attitudes was a recurring theme in participant statements.

*“Negative social attitudes towards us and our clients intensified during the COVID-19 pandemic compared to before. This was evident in landlords refusing to renew housing leases*,* and our families being unwilling for us to continue working at harm reduction centers*,* viewing us as carriers of the virus. I wish these attitudes would change.” (Participant 6)*.

#### Enhancing professional development for social workers

The COVID-19 pandemic highlighted the increasing isolation of the social work profession. Participants argued that, unlike other service professions, social work has not evolved to adequately address challenges like staff shortages and limited recognition in academic and social spheres, particularly during crises.

*“The COVID-19 pandemic has negatively impacted the social work profession*,* leading to decreased interest among new entrants and a reluctance to pursue further education among experienced professionals. Policymakers must prioritize strategies to revitalize and strengthen the social work field.” (Participant 8)*.

### Educational needs

The COVID-19 pandemic highlighted the urgent need for comprehensive training among clients of harm reduction services, who often come from marginalized populations. Participants emphasized the importance of “developing creative training approaches” and “conducting rigorous needs assessments” to tailor educational programs to the specific needs of this population.

#### Developing creative training approaches

During the COVID-19 pandemic, traditional educational methods for teaching personal hygiene and adherence to health protocols were found to be inadequate due to varying levels of literacy and comprehension among clients. Consequently, participants highlighted the need for more innovative and up-to-date educational approaches.

*“Our clients*,* who engage in high-risk behaviors*,* often disregarded conventional training aimed at reducing social interactions during the COVID-19 pandemic. I believe that employing creative educational methods*,* such as showcasing a video depicting the potential consequences of these interactions*,* could have been more effective.” (Participant 8)*.

#### Conducting rigorous needs assessments

Participants emphasized the increased need for thorough assessments of their needs, especially during the COVID-19 pandemic, more so than before.

*“During the pandemic*,* we had to frequently adapt to changing health guidelines. It would have been more beneficial if trainings had been specifically tailored to address our health needs and concerns as healthcare workers in the context of the evolving pandemic”. (Participant 5)*

*“In my opinion*,* the COVID-19 health protocols were not suitable for our needs. I wish they had been designed based on an assessment of the centers’ needs.” (Participant 8)*.

### Communication needs

Due to mandatory social distancing and communication restrictions during the COVID-19 pandemic, participants increasingly recognized the need to address communication needs. In this regard, they identified two key subcategories: “behavioral therapy” and “communication tools “.

#### Behavioral therapy

Participants noted the need for behavioral therapy for their clients, as unhealthy behaviors among clients of their women’s harm reduction centers had increased during the COVID-19 pandemic, disrupting their service delivery.

*“A significant portion of our clients are sex workers. During the COVID-19 pandemic*,* they exhibited multiple risky sexual behaviors*,* which made the need for behavioral interventions even more critical for them.” (Participant 2)*.

*“The COVID-19 highlighted the need for behavioral change among clients*,* particularly those who engaged in group drug use*,* which was considered a dangerous situation during that time.”*

*(Participant 4)*.

#### Communication tools

Participants emphasized the crucial role of utilizing various communication tools more than ever during the COVID-19 pandemic. The need to establish two-way communication using phone calls, text messages, and video conferencing platforms became significantly more important for following up with and educating clients.

*“The COVID-19 pandemic exposed the limitations of our traditional communication strategies in supporting clients with substance use disorders. As attendance rates decreased due to social distancing*,* it became evident that alternative communication methods were necessary to effectively engage clients and facilitate recovery. “(Participant 5)*.

In summary, participant feedback demonstrated that the COVID-19 pandemic made the needs of service providers in harm reduction centers more acute. Certain of these needs took on a critical nature during this period, emphasizing the increased importance of their resolution.

## Discussion

This research explored the changing needs of service providers in women’s harm reduction centers in Tehran, Khuzestan, and Kermanshah during the COVID-19 pandemic. The study revealed several important and developing needs highlighted by the participants, spanning financial, regulatory, structural, infrastructural, sociocultural, educational, and communication areas. Meeting these identified needs is essential to ensure the ongoing delivery of effective support within these centers, especially when considering potential future crises.

Our findings underscore the critical role of financial stability in mitigating the impact of public health crises on vulnerable populations. Participants in our study consistently identified financial constraints as the most pressing need during the COVID-19 pandemic, emphasizing the urgent need for measures such as removing credit restrictions and controlling inflation. While this aligns with the broader economic impact on healthcare facilities globally [22], where financial strain led to resource shortages and compromised care quality, our research from the specific context of women’s harm reduction centers in Tehran, Khuzestan, and Kermanshah reveals a critical policy gap: the direct threat to the accessibility and continuity of vital harm reduction services for a particularly marginalized group. This is echoed by studies in Canada [23] and globally [24] highlighting the disproportionate financial burden on vulnerable populations, with Russell et al. (2021) specifically noting the reduced access for women who use drugs. However, our findings extend this by demonstrating removing credit limits, and controlling inflation which necessitates targeted policy interventions. The experiences of our participants, mirroring the policy concerns raised in the USA [25], underscore that policymakers must proactively consider the financial sustainability of these centers through mechanisms like establishing dedicated emergency funding or flexible grant programs. The global survey by Radfar et al. (2021) supports the need for financial investment [26]. Our study adds a crucial localized perspective, highlighting how the lack of tailored financial support directly jeopardizes the ability to retain staff or procure essential supplies for a highly vulnerable population during public health emergencies. Therefore, policy recommendations should prioritize the development of specific financial resilience strategies for women’s harm reduction centers in Tehran, Khuzestan, and Kermanshah, ensuring they are not disproportionately affected by economic downturns during crises. This could involve integrating harm reduction funding into mainstream healthcare budgets or providing specific financial incentives for centers serving marginalized populations.

While this study underscores the crucial need for continuous monitoring and reevaluation of performance metrics during crises like the COVID-19 pandemic, the implications extend beyond mere observation. Building on the findings of Rahmani et al. (2024), which highlighted supervisory capacities as pivotal in bolstering the responsiveness of women’s harm reduction centers through improvements in COVID-19 registry, staff vaccinations, and client screening [27], our results suggest a critical role for strengthening managerial oversight as a policy lever. This is further supported by the Australian experience, where sustained monitoring, as noted by Roxburgh et al. (2021), ensured the adaptability of harm reduction services [28]. The divergence in perspectives regarding the importance of monitoring and inspection, both within our findings and in limited existing research, points to a policy gap requiring more in-depth investigation. Crucially, the review by Wilkinson et al. (2020), which documented significant disruptions or closures of harm reduction services, underscores the potential of enhanced monitoring and inspection mechanisms, implemented as policy mandates, to proactively mitigate service gaps and ensure consistent access to vital support during public health emergencies [29]. Moving forward, policy should prioritize the integration of robust monitoring frameworks with clear supervisory responsibilities to enhance the resilience and adaptability of harm reduction services in the face of future crises.

Participants in our study identified ‘structural needs’– including the co-location of shelters and DICs, increased staffing, flexible regulations, clear job descriptions, improved accommodations, and adequate equipment– as critical areas for improvement. These findings, while echoing the significance of systemic failures in exacerbating overdose mortality during the COVID-19 pandemic as highlighted by Conway et al. (2022) [30], point directly to actionable policy changes. To mitigate the challenges identified, policy must prioritize structural and resource-based interventions. For instance, the co-location of services, as called for by participants, could enhance service accessibility and coordination. Furthermore, the staffing shortages reported necessitate policy-level commitments to increased funding and workforce development initiatives. The constraints on harm reduction service provision during the pandemic, as documented by Brener et al. (2021) [31], underscore the urgent need for policies that ensure the resilience of these services during crises. This requires proactively addressing structural inequalities and social exclusion, as Brener et al. (2021) suggest, not just to minimize immediate harms and enhance vaccination rates, but also to build a more robust long-term prevention infrastructure. The impact of COVID-19-induced structural and societal disruptions on individual mental health and substance use, as evidenced in the U.S. study by Walters et al. (2022) [32], further reinforces the need for policy interventions that address these broader determinants of health. This includes investing in social support systems and mental health services alongside harm reduction efforts. Ultimately, the consistent emphasis on structural issues as a driver of mortality among vulnerable populations, a point also stressed by McNeil et al. (2022) in the United States [33], compels healthcare planners and policymakers to move beyond individual-focused interventions and prioritize systemic changes that can create a more supportive and accessible environment for harm reduction services.

Our study identified ‘civil needs’– specifically ensuring employee safety and effective judicial protection– as crucial considerations for harm reduction service provision. This finding, when considered alongside the impact of criminal-legal procedure changes on HIV-risk behaviors among women who use drugs during the COVID-19 pandemic Smoyer et al. (2024) [34], underscores the significant influence of the legal environment on the well-being and health behaviors of this population. To address these civil needs effectively, policies must proactively safeguard both service providers and clients within the legal framework. The findings of Sun et al. (2022) [35], which documented infringements on civil and political rights during COVID-19 emergency orders, such as freedom of movement and the right to a fair trial, directly highlight the potential for public health crises to erode fundamental rights. This alignment with the concerns of harm reduction providers in our study suggests a critical policy imperative: future crisis responses must integrate robust safeguards for civil liberties. This necessitates a careful balancing act, ensuring public health measures do not unduly restrict individual freedoms or compromise access to justice. The broader impact of the COVID-19 pandemic on national and international legal and civil frameworks further emphasizes the need for policymakers to anticipate and mitigate these impacts in future crises through the development of clear legal guidelines and protections that are resilient even during public health emergencie [36]. Moving forward, policy development must explicitly consider the civil rights implications of public health measures to ensure the safety and legal protection of both those providing and those accessing essential harm reduction services.

Our study identified significant ‘social-cultural needs,’ specifically the imperative to change negative social attitudes and enhance professional development for social workers in the harm reduction field. These findings, when considered alongside the persistent stigma faced by people who use drugs during the COVID-19 pandemic‌ [37], highlight a critical policy barrier to effectively addressing the health and safety of this vulnerable population. The public opposition to allocating life-saving resources, stemming from this stigma, directly impacts the feasibility and effectiveness of harm reduction efforts. To counter this, policy interventions must actively target stigma reduction through public health campaigns and educational initiatives. Furthermore, the barriers to engaging people who use drugs in harm reduction services during the pandemic, as reported by Austin et al. (2022) [38] including social isolation, reduced access, and limited engagement, underscore the need for policies that proactively outreach to and build trust with this community. This requires investing in community-based outreach programs and adapting service delivery models to overcome these social-cultural barriers. The exacerbation of stigma and discrimination during the COVID-19 pandemic, as linked to negative outcomes like increased overdose deaths and limited healthcare access [39], further emphasizes the urgency of policy changes that address these deep-seated social-cultural issues. Moving forward, policy should prioritize funding and implementing comprehensive anti-stigma campaigns and invest in the professional development of social workers to equip them with the skills to navigate and challenge negative social attitudes effectively. This dual approach is crucial for creating a more supportive and accessible environment for harm reduction services and ultimately improving outcomes for people who use drugs.

Our study identified significant ‘educational needs,’ specifically the development of creative training approaches and communication tools within harm reduction services. This finding, when considered alongside the innovative adaptations to opioid use disorder (OUD) treatment and harm reduction services during the COVID-19 pandemic highlighted by Krawczyk et al. (2021) [40], such as telehealth and expanded medication access, suggests a crucial policy direction: the integration of flexible and technology-driven educational models within standard service delivery. The success of these adaptations in maintaining and even improving service access during a crisis underscores their potential for broader application in enhancing the accessibility and reach of educational initiatives for vulnerable populations. Furthermore, Nguyen et al. (2022) [41] findings on the persistence of group methamphetamine use and unsafe sex as HIV/HCV risk factors among people who inject drugs during lockdowns point to a critical policy gap in sustained public health interventions. This necessitates the development and implementation of comprehensive and culturally sensitive educational programs specifically targeting these behaviors, particularly among disproportionately impacted groups like sex workers. The consistent emphasis on ‘education and outreach’ as a core element in pandemic and outbreak responses for homeless populations [1] and the importance of education and awareness for both homeless individuals and service providers during COVID-19 [7] collectively underscore a key policy imperative: investing in accessible, innovative, and targeted educational strategies is essential for mitigating health risks and improving outcomes for vulnerable populations during and after public health emergencies. Moving forward, policy should prioritize funding for the development and dissemination of creative educational tools and training programs that leverage technology and adapt to the specific needs and contexts of the populations being served.

Our study underscores the indispensable role of effective communication as a cornerstone of resilient service delivery, particularly when navigating the complexities of crises such as the COVID-19 pandemic. This finding, supported by Picchio et al. (2020) [3] demonstration of communication’s crucial role in securing resources and maintaining service continuity during the pandemic, points to a clear policy imperative: investing in robust communication infrastructure and protocols within service organizations. Furthermore, the challenges highlighted by Nguyen et al. (2022) [41] regarding the prevalence of group methamphetamine use as a coping mechanism during lockdowns underscore the need for policies that prioritize open communication and accessible support networks. This suggests that public health strategies must actively promote communication channels to mitigate the negative impacts of social isolation and stress, thereby addressing risky group behaviors. Building on this, Nyamathi et al. (2022) [10] findings on the importance of maintaining social connections as a coping mechanism during the pandemic suggest a policy focus on leveraging communication technologies and community outreach to foster these connections, thereby safeguarding mental and emotional well-being during crises. The persistent barriers to service delivery identified by Austin et al. (2022) [38] including isolation, reduced access, and limited engagement– further emphasize that effective communication strategies are not merely supportive but are a critical policy lever for overcoming these obstacles. Policies should therefore mandate and fund the development and implementation of comprehensive communication strategies that facilitate information sharing, build trust, and strengthen relationships between service providers, clients, and the broader community, ensuring the continued delivery of essential harm reduction services even amidst crisis-induced disruptions.

In conclusion, the findings of this study, in conjunction with related research, highlight the significant and multifaceted impact of the COVID-19 pandemic on individuals, organizations, societies, and political systems, creating numerous new needs that necessitate comprehensive and coordinated governmental responses. While the multi-centered design, strategically selecting harm reduction centers from three diverse Iranian provinces (Tehran, Khuzestan, and Kermanshah) with varying population densities and socio-economic conditions, offered a valuable perspective across diverse contexts, several methodological constraints warrant consideration. Firstly, the reliance on self-reported data from service providers introduces the potential for participant bias, including social desirability bias or recall inaccuracies regarding their experiences and observations. Secondly, while the inclusion of three distinct provinces enhanced regional diversity, the study’s scope was limited to these specific areas, potentially overlooking unique challenges and adaptations in other regions of Iran, thus representing a regional limitation. Finally, the qualitative nature of data collection, while rich in detail, inherently presents limitations in capturing the full spectrum of experiences and may have resulted in gaps in data collection regarding specific quantitative aspects of service delivery changes or the precise scale of certain challenges reported. Acknowledging these methodological constraints provides a more nuanced understanding of the study’s findings and informs future research directions that could employ quantitative methods or expand geographical representation to further validate and generalize these insights.

## Conclusion

This study compellingly highlights the significantly increased demands and multifaceted challenges faced by service providers in women’s harm reduction centers during the COVID-19 pandemic, underscoring the critical need for resilient service continuity in future crises. Our findings offer actionable insights for community health managers in resource allocation and targeted support. To further strengthen the field, future research should comparatively examine service delivery models across different settings (men’s centers, addiction facilities) and rigorously evaluate the effectiveness of public health policies supporting harm reduction, particularly concerning workforce well-being and interagency collaboration. Policy should prioritize funding standardized crisis preparedness plans, implementing mental health support for providers, and establishing clear interagency protocols to build a more robust and equitable harm reduction system capable of effectively responding to future public health challenges.

## Data Availability

The data obtained through interviews contains personal and sensitive information from the participants. Due to ethical considerations and our commitment to protecting their privacy, the complete dataset is not publicly available.

## Declarations

### Regarding AI Use

The authors employed Google Gemini as a tool to refine the language and improve the clarity of this manuscript. The final content remains the sole responsibility of the authors.

## Abbreviations

HIV: Human immunodeficiency virus
PPE: Personal protective equipment
DICs: Drop-in Centers
